# Transcutaneous bilirubin estimation in extremely low birth weight infants receiving phototherapy: a prospective observational study

**DOI:** 10.1186/s12887-018-1207-7

**Published:** 2018-07-10

**Authors:** Vidit Bhargava, Daniel Tawfik, Bruce Niebuhr, Sunil K. Jain

**Affiliations:** 1 Department of Pediatrics, Division of Pediatric Critical Care, Lucile Salter Packard Children’s Hospital, 770 Welch Road, Suite 435, Palo Alto, CA 94304 USA; 2 Department of Pediatrics, University of Texas Medical Branch, Galveston, TX USA; 3 Department of Pediatrics, Division of Neonatology, University of Texas Medical Branch, Galveston, TX USA

**Keywords:** Hyperbilirubinemia, ELBW, Kernicterus, Transcutaneous bilirubinometry

## Abstract

**Background:**

Measurement of transcutaneous bilirubin (TcB) is a quick, reliable and painless method to guide management of hyperbilirubinemia. Studies in term and late preterm infants have found that TcB measurements from covered areas (TcB-C) during phototherapy (PHT) co-relate well with serum bilirubin levels. Limited data exists in extremely low birth weight (ELBW) infants.

**Methods:**

In this prospective observational study, an opaque patch was placed on the back of an ELBW infant prior to initiation of PHT. TcB-C and TcB-E (TcB from exposed area) levels were measured at birth and at 24-h intervals for 5 days. Total serum bilirubin (TSB) levels were also measured within 30 min of obtaining TcB levels. A Wilcoxon signed rank test was used for data analysis. A mixed effect model was used to adjust for repeated measurements over time. The *p* value < 0.05 was considered significant.

**Results:**

A total of 19 infants were enrolled in the study, with a mean gestational age of 26 ± 2 weeks and mean weight 827 ± 127 g. The difference between TcB-C and TSB was 2.68 ± 2.41 mg/dl (mean ± SD, *p* <  0.001). In contrast, the difference between TcB-E and TSB was − 0.51 ± 1.74 mg/dl (*p* = 0.02). TcB-C consistently overestimates TSB, while TcB-E consistently underestimates TSB.

**Conclusions:**

During PHT exposure, TcB-C does not correlate with TSB values in ELBW infants. TcB-C levels cannot be used as a surrogate for TSB measurement in ELBW infants.

## Background

Hyperbilirubinemia is seen in almost two-thirds of term and more than two-thirds of all preterm infants [1]. The incidence of kernicterus has dramatically decreased since the onset of regular screening and aggressive management with phototherapy (PHT). In one post-mortem series of premature infants, kernicterus was found to be virtually non-existent [2]. The USA Kernicterus Registry reported 125 infants ≥35 weeks estimated gestational age with kernicterus/acute bilirubin encephalopathy between 1992 to 2004. No specific serum bilirubin values coincided with onset of kernicterus in these infants [3]. Hence, while the incidence has substantially decreased, a considerable disease burden still exists in term and preterm infants with hyperbilirubinemia. The American Academy of Pediatrics Subcommittee on Hyperbilirubinemia recommends that every infant be screened for hyperbilirubinemia by TSB or TcB at 24 h of life and with subsequent measurements guided by the bilirubin level at 24 h of life and the presence of other risk factors [4]. A recent NICHD Neonatal Research Network study focused exclusively on the management of hyperbilirubinemia in ELBW infants demonstrated that aggressive management of hyperbilirubinemia with PHT demonstrated significant benefits in neurodevelopmental outcomes [5].

Transcutaneous bilirubinometry is a quick, painless and reliable alternative to serum bilirubin measurements in the management of hyperbilirubinemia [6]. Following PHT, TcB measurements are considered unreliable, as PHT causes bleaching of the skin [7]. We and others have identified a moderate correlation between TcB measurements taken from skin covered by an opaque patch and TSB levels in term and late preterm infants following phototherapy [8, 9]; however, there is little data on the reliability of TcB-C in ELBW infants. We aim to investigate if TcB-C following PHT exposure is a suitable surrogate for TSB in ELBW infants.

## Methods

A prospective observational study was performed in NICU at University of Texas Medical Branch (UTMB) at Galveston after approval from the local institutional review board (IRB). Subjects were enrolled in the study from January 2014 to June 2016. The study population consisted of ELBW infants born at UTMB receiving phototherapy for hyperbilirubinemia. ELBW infants were defined as infants with a birth weight of less than 1000 g. Following IRB approval, subjects were enrolled in the study with parental assent. The requirement for written consent was waved by the IRB at our institution. In our NICU, all ELBW infants get prophylactic PHT for first 5 days. All ELBW infants were eligible for the study. Infants with congenital viral infections, conjugated hyperbilirubinemia, sepsis, major congenital malformations, ABO/Rh incompatibility or gastrointestinal illness were excluded from the study.

**Fig. 1:**
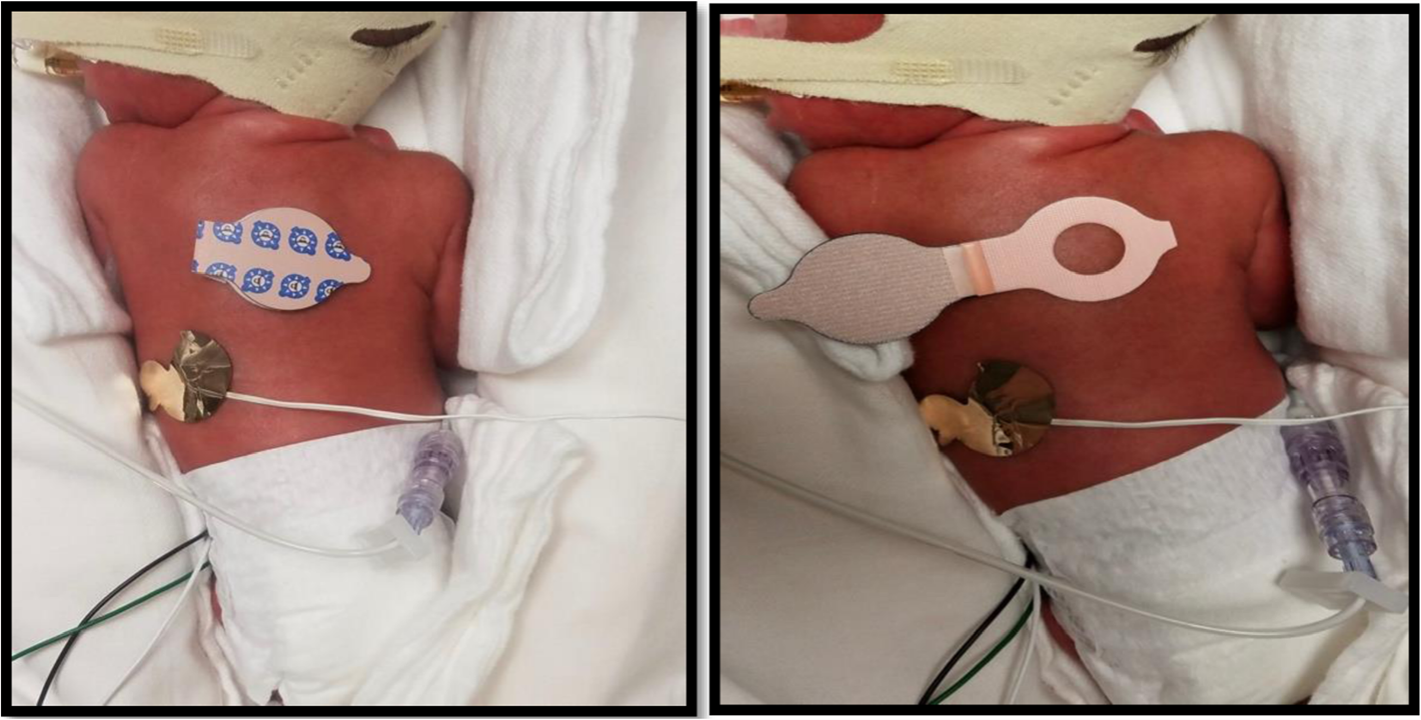
Demonstrating photo-opaque patch placement in an ELBW neonate

Prior to starting PHT, an opaque patch was placed on the back of the infant. (Fig. 1) The forehead could not be used to place the patch as most of these infants required CPAP. After placing the opaque patch, PHT was started by using a Giraffe Blue Lite PT system (GE Healthcare, Chicago, IL) and continued for 5 days. TcB-C, TcB-E and TSB levels were obtained at birth and every 24 h for 5 days by trained NICU nurses. TcB-C was obtained from the skin covered by the opaque patch. TcB-E was obtained from a site not covered by the patch but adjacent to it. TcB measurements were obtained using the Respironics™ BiliCheck noninvasive bilirubin meter. Clinical data was collected from the electronic medical record (EMR) and included gestational age, birth weight, sex, ethnicity, blood group, maternal blood group, and ABO/Rh incompatibility, if any.

Serum bilirubin levels (TSB) were used as the gold standard. The required sample size for each method was calculated using a power analysis. The power analysis indicated that 32 subjects (16 in each group) were needed to have 80% power for detecting a medium-sized effect (± 0.5 mg/dl) with *p* <  0.05 for statistical significance. A Wilcoxon signed rank test was used to compare the means of the absolute difference between transcutaneous and serum bilirubin measurements for each patient at each time point (24, 48, 72 and 96 h). The absolute difference between transcutaneous and serum bilirubin measurements for each patient is shown on a Bland-Altman plot for easy visualization. Analysis was performed using a mixed effect model to account for repeated measures over time. All analysis was done using JMP® Pro software.

## Results

**Fig. 2:**
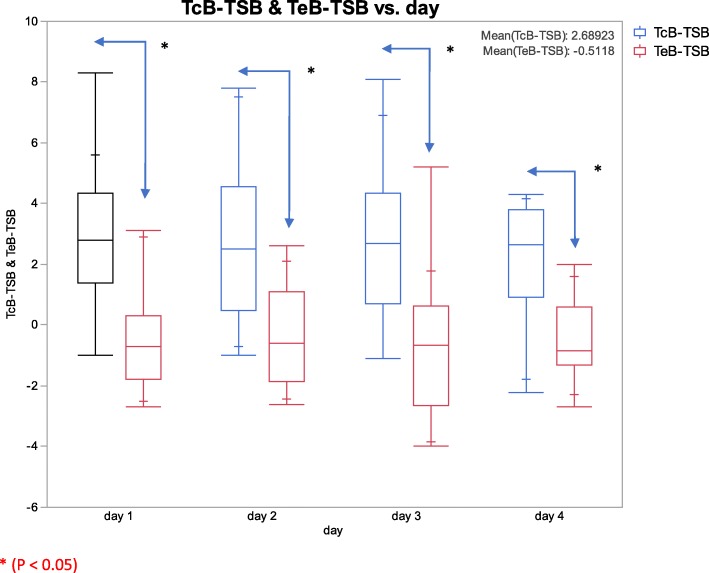
Graph depicting the difference between TcB-C or TcB-E and TSB on various days

Twenty subjects were initially enrolled in the study. One subject was excluded from the study due to neonatal demise at day 2 of life. The mean birth weight was 827 ± 127 g, and the mean estimated gestational age was 26 ±2 weeks. Caucasians, Hispanics and African-Americans constituted the majority of study group participants. Patient demographics are shown in Table 1. Differences between TcB-C or TcB-E and TSB are shown in Fig. 2.

**Table 1:** Patient demographics

Sample size	19
Males	13 (68%)
Females	6 (32%)
Gestational age (in weeks)	26 ± 2
Birth weight (in grams)	827 ± 127 g
Race	19 (100%)
Caucasian	6 (31.6%)
African-American	3 (15.8%)
Hispanic	8 (42.1%)
Other	2 (10.5%)

**Fig. 3:**
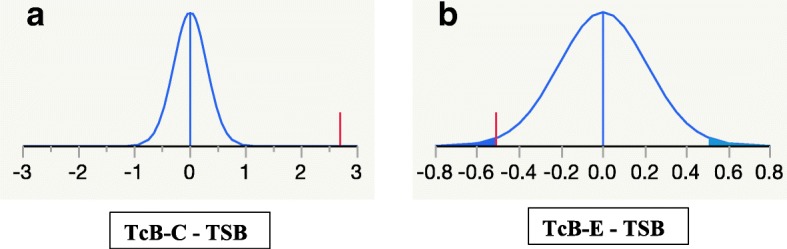
**a** The probability density function and the mean of difference between the TcB-C and TSB. **b** The probability density function and the mean of difference between the TcB-E and TSB. The vertical bar represents the mean compared to the normal distribution

**Fig. 4:**
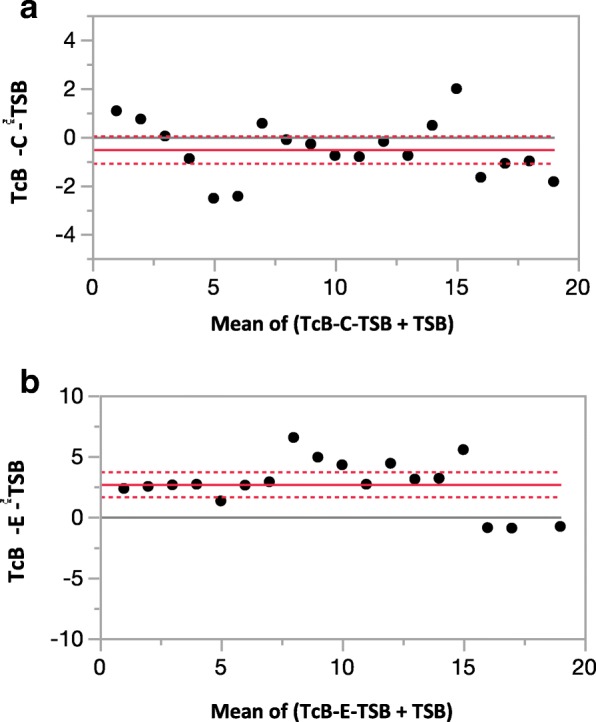
**a** A Bland Altman plot depicting the difference between TcB-C and TSB for each patient. **b** A Bland Altman plot depicting the difference between TcB-E and TSB for each patient

A Wilcoxon signed rank test was used to compare the TcB-C or TcB-E to TSB. The mean of difference between TcB-C and TSB was 2.68 ± 2.41 mg/dl (*p* < 0.001, IQR 1.1–4.2) mg/dl. In contrast, the mean of difference between TcB-E and TSB was − 0.51 ± 1.74 mg/dl (*p* = 0.02, IQR -1.77 – 0.47, shown in Table 2 and Fig. 3) The absolute differences between the TcB-C or TcB-E and TSB for each patient were like the trends seen in the Wilcoxon signed rank test and are depicted as Bland Altman plots for easy visualization (Fig. 4). TcB-C overestimated serum bilirubin levels for most patients with a difference of 2.71 ± 0.49 mg/dl (*p* < 0.001), while TcB-E underestimated serum bilirubin levels with a difference of − 0.51 ± 0.26 mg/dl (*p* = 0.07).

**Table 2:** Serum (TSB) and transcutaneous bilirubin measurements (TcB-C/TcB-E)

	Mean	SD	IQ range (25–75%)	SEM	*P* value
TcB-C – TSB	2.68	2.41	1.1–4.2	0.29	< 0.001
TcB-E – TSB	−0.51	1.74	−1.77 – 0.47	0.21	0.02

Similar results were obtained following adjustment of the bilirubin estimation for repeated measures over time. There was no effect of randomization of the day of bilirubin estimation on the overall results. The TcB-C continued to overestimate the TSB (*p* < 0.001), while TcB-E continued to underestimate the TSB (*p* < 0.001). However, when the bilirubin estimation was randomized for each patient the overall model improved by 18.9% and this difference was found to be significant (*p* = 0.02).

Thus, TcB-C overestimates the estimation of bilirubin compared to serum bilirubin, while TcB-E underestimates the estimation of bilirubin compared to serum bilirubin. Hence, TcB-C and TcB-E may not be used as surrogates for bilirubin estimation in ELBW infants receiving phototherapy.

## Discussion

We evaluated the role of TcB estimation in ELBW infants who were receiving PHT. We found TcB-C did not correlate well with TSB levels in ELBW infants and, in fact, were often higher than TSB. Within 24 h of PHT exposure, TSB values decreased significantly compared to the TcB-C values and thereafter the values decline gradually. Skin exposure to PHT converts unconjugated bilirubin into its water-soluble isomer by photoisomerisation. There is a continuous and bidirectional movement of bilirubin isomers between the blood and the skin leading to skin bleaching [10]. During PHT exposure, TcB drops due to skin bleaching. But if a part of the skin is covered by a photo-opaque patch bleaching will be minimal. The lateral movement of bilirubin from exposed to the covered areas of skin is also minimal, thus accounting for the higher bilirubin levels in the covered areas of skin [11]. This finding in our study is similar to that reported by by Ozkan, et al., who noted that TcB-C was slower to decline compared to TSB and TcB-E values. In their study, TcB-E levels declined rapidly in the first 6 h after starting PHT, while the decline in TcB-C was not noticed until 12 h after starting PHT. Serum bilirubin values were noted to decline gradually during the study period.

In studies performed in term and preterm infants, the TcB-C levels were found to be comparable with serum bilirubin levels [8]. This was likely due to a gradual decline in TSB levels, permitting equilibration of TcB-C with TSB. However, in our study TSB levels were significantly lower than TcB-C levels. This significant decline in TSB in ELBW infants is likely multifactorial in nature. First, bilirubin clearance depends on a multiple factors including wavelength of light used for PHT, irradiance of light, skin surface area exposed to PHT, and rates at which bilirubin is removed from skin and blood [12]. Stratum corneum in ELBW is immature [13], allowing rapid clearance of bilirubin from exposed areas of the skin (bleaching effect) leading to a significant decline in TcB-E levels. Second, since bilirubin levels in the skin rapidly decline following initiation of phototherapy, equilibration between the skin and serum leads to a rapid decline in serum bilirubin levels.

Our study has a few limitations. The sample size of the study population was small and the study was not adequately powered to reliably predict secondary outcomes such as the correlation between TSB and TcB-E values. We measured serum bilirubin at 24-h intervals which may have affected the micro trends in bilirubin levels via different methods during the first 24 h. More frequent bilirubin sampling may be helpful in delineating dermal bilirubin kinetics in ELBW infants.

## Conclusions

We suggest that TcB-C is not be a helpful surrogate for TSB in ELBW infants receiving PHT. Studies in larger cohorts may be needed to further substantiate these findings. In the future, we suggest measuring TcB and TSB at shorter intervals and in larger cohorts to evaluate the dermal bilirubin kinetics.

## Abbreviations

NICU: Neonatal intensive care unit
PHT: Phototherapy
TcB-C: Transcutaneous bilirubin estimation from covered areas of skin
TcB-E: Transcutaneous bilirubin estimation from exposed areas of skin
TSB: Total serum bilirubin
UTMB: University of Texas Medical Branch, Galveston
